# *Naegleria fowleri* Cathepsin B Induces a Pro-Inflammatory Immune Response in BV-2 Microglial Cells via NF-κB and AP-1 Dependent-MAPK Signaling Pathway

**DOI:** 10.3390/ijms23158388

**Published:** 2022-07-29

**Authors:** Hương Giang Lê, Jung-Mi Kang, Tuấn Cường Võ, Byoung-Kuk Na

**Affiliations:** 1 Department of Parasitology and Tropical Medicine, and Institute of Health Sciences, Gyeongsang National University College of Medicine, Jinju 52727, Korea; gianglee291994@gmail.com (H.G.L.); jmkang@gnu.ac.kr (J.-M.K.); vtcuong241@gmail.com (T.C.V.); 2 Department of Convergence Medical Science, Gyeongsang National University, Jinju 52727, Korea

**Keywords:** *Naegleria fowleri*, cathepsin B cysteine protease, microglial cells, pro-inflammatory response, MAPK, NF-κB

## Abstract

*Naegleria fowleri* is a ubiquitous protozoa parasite that can cause primary amoebic meningoencephalitis (PAM), a fatal brain infection in humans. Cathepsin Bs of *N. fowleri* (NfCBs) are multifamily enzymes. Although their pathogenic mechanism in PAM is not clearly understood yet, NfCBs have been proposed as pathogenic factors involved in the pathogenicity of amoeba. In this study, the immune response of BV-2 microglial cells induced by NfCB was analyzed. Recombinant NfCB (rNfCB) evoked enhanced expressions of TLR-2, TLR-4, and MyD88 in BV-2 microglial cells. This enzyme also induced an elevated production of several pro-inflammatory cytokines such as TNF-α, IL-1α, IL-1β, and IL-6 and iNOS in cells. The inhibition of mitogen-activated protein kinases (MAPKs), including JNK, p38, and ERK, effectively reduced the production of these pro-inflammatory cytokines. The rNfCB-induced production of pro-inflammatory cytokines in BV-2 microglial cells was suppressed by inhibiting NF-kB and AP-1. Phosphorylation and nuclear translocation of p65 in cells were also enhanced by rNfCB. These results suggest that NfCB can induce a pro-inflammatory immune response in BV-2 microglial cells via the NF-κB- and AP-1-dependent MAPK signaling pathways. Such a NfCB-induced pro-inflammatory immune response in BV-2 microglial cells might contribute to the pathogenesis of PAM caused by amoeba, by exacerbating deleterious immune responses and tissue damages in *N. fowleri*-infected foci of the brain.

## 1. Introduction

*Naegleria fowleri*, commonly known as a brain-eating amoeba, is a pathogen causing primary amoebic meningoencephalitis (PAM) in humans. When actively proliferating trophozoites of this amoeba infect humans via a nasal route, the amoeba can penetrate the nasal mucosa, migrate to the brain via olfactory nerves, and induce fatal pathologic events in the central nervous system (CNS) [1]. This amoeba can cause extensive damage in the brain, characterized by acute hemorrhagic inflammation, resulting in death within 7–10 days of the infection. In the early phase of *N. fowleri* infection, the host’s innate immune system is activated to secrete mucin that can inhibit the adherence of amoeba to host cells and protect host cells [2]. Once the amoeba reaches the brain by overcoming the initial immune response of the host, intense inflammatory responses contributing to tissue damage occur, resulting in massive hemorrhage and the lytic necrosis of leukocytes and brain tissues [3]. Regarding how the amoeba induces host cell death and the inflammation response of the hosts, two primary pathogenic mechanisms have been proposed: contact-dependent and contact-independent mechanisms [4]. Direct contact, followed by the destruction of host cells by *N. fowleri* trophozoites via active trogocytosis is likely to be the major pathogenic event caused by the amoeba [4]. Meanwhile, the contact-independent mechanism is an indirect pathogenic event, mainly caused by diverse secreted or released proteins and cytolytic factors from the amoeba [5,6,7].

Cysteine proteases of pathogenic protozoan parasites play pivotal roles in the biology and pathogenicity of parasites [8]. They are essentially involved in diverse processes, including the invasion, nutrition, development, pathogenesis, and the survival of parasitic protozoa, by mediating essential biological events of parasites, as well as modulating host immune responses [9,10,11,12,13]. Cathepsin B family cysteine proteases of *N. fowleri* are secretory proteins that are likely to be involved in the pathogenicity of amoeba by facilitating the invasion of the amoeba and modulating host immune responses [14,15]. However, the biological roles of these enzymes and their functional contributions to PAM have not been clearly understood yet. In order to extend our understanding of the biological functions of the cathepsin B enzymes of *N. fowleri* associated with pathological events in PAM, it is necessary to investigate the underlying molecular mechanisms of these enzymes associated with host immune response.

In the present study, the immune response of BV-2 microglial cells induced by a cathepsin B of *N. fowleri* (NfCB) was analyzed. Recombinant NfCB induced a pro-inflammatory immune response in BV-2 microglial cells by promoting the production of pro-inflammatory cytokines, including TNF-α, IL-1α, IL-1β, and IL-6 via the MyD88-dependent TLR-2/TLR-4 pathway. This inflammatory response of BV-2 microglial cells was regulated by mitogen-activated protein kinases (MAPKs) and NF-κB/AP-1 signaling pathways. These results suggest that NfCB can induce a pro-inflammatory immune response in BV-2 microglial cells, eventually contributing to the pathogenesis of PAM by exacerbating deleterious inflammatory responses and tissue damage in *N. fowleri*-infected foci of the brain.

## 2. Materials and Methods

### 2.1. Preparation of Recombinant NfCB (rNfCB)

The rNfCB was produced in *Escherichia coli* as described previously [15]. Purified rNfCB was refolded in optimized refolding buffer, activated at an acidic pH, and concentrated using a Centriprep (10 kDa cut-off; Merck Millipore, Burlington, MA, USA). The enzyme activity of rNfCB was measured with benzyloxycarbonyl-_L_-leucyl-_L_-arginine 4-methyl-coumaryl-7-amide (Z-LR-MCA; Peptide International, Louisville, KY, USA) [15]. To remove lipopolysaccharide (LPS) that might potentially contaminate the rNfCB, a Detoxi-gel endotoxin removing column (Thermo Fischer Scientific, Waltham, MA, USA) was used following the manufacturer’s protocols. No detectable amount of residual endotoxin in the rNfCB was confirmed with a Chromogenic Endotoxin Quant Kit (Thermo Fisher Scientific, Waltham, MA, USA). LPS-depleted rNfCB was filtered with a sterile syringe filter (0.22 µM; Merck Millipore, Burlington, MA, USA) and was used for all cell-based experiments. The purity and concentration of rNfCB were analyzed via 12% sodium dodecyl sulfate–polyacrylamide gel electrophoresis (SDS–PAGE) and a BCA protein assay kit (Thermo Fischer Scientific, Waltham, MA, USA), respectively. Inactive rNfCB was prepared by heating the enzyme at 60 °C for 6 h. Complete loss of enzyme activity of heat-inactivated rNfCB was confirmed using an enzyme activity assay, using Z-LR-MCA (Peptide International, Louisville, KY, USA).

### 2.2. Cultivation of BV-2 Microglial Cells and Treatment of rNfCB

BV-2 microglial cells were cultured in Dulbecco’s Modified Eagle’s Medium (DMEM; Welgene, Daegu, Korea) supplemented with 10% heat-inactivated fetal bovine serum (FBS; Gibco, Grand Island, NY, USA) and 1% penicillin/streptomycin (Gibco, Grand Island, NY, USA). The cells were incubated at 37 °C in a humidified incubator under a 5% CO_2_ atmosphere. The potential cytotoxicity of rNfCB to BV-2 microglial cells was analyzed using a cell viability assay prior to experiments. BV-2 microglial cells were seeded into a 96-well microplate (2 × 10^4^ cells/well) and incubated at 37 °C with 5% CO_2_ overnight until 70% confluence. Serially diluted rNfCB (0, 5, 10, 15, 20, 40, 50, 100, and 150 µg/mL) was added to cells and incubated for 24 h. Cell viability was analyzed using a CellTiter-Blue^®^ Cell Viability Assay (Promega, Madison, WI, USA) according to the manufacturer’s instructions.

### 2.3. Analysis of the Pro-Inflammatory Immune Response in BV-2 Microglial Cells Induced by rNfCB

To analyze the effect of rNfCB on the production of pro-inflammatory cytokines in BV-2 microglial cells, 10^5^ cells were seeded in each well of a 12-well plate (Thermo Fischer Scientific, Waltham, MA, USA) and incubated until 70% confluence. After changing the media to fresh serum-free media, cells were incubated with different concentrations (20 µg/mL and 100 µg/mL) of active or heat-inactivated rNfCB, respectively, for 6 h. To observe the overall immune responses in BV-2 microglial cells upon treatment with rNfCB, a cytokine array assay was performed. The supernatants from the cells with or without treatment with rNfCB (100 µg/mL) were collected, and expression profiles of cytokines and chemokines were analyzed with Proteome Profiler^TM^ Mouse Cytokine Array Panel A (R&D systems, Minneapolis, MN, USA), following the manufacturer’s protocols. For further analysis, total RNA was isolated from the cells using RNAiso Plus (Takara, Otsu, Japan) following the manufacturer’s instructions. Purified total RNA was digested with RNase-free DNase (Takara, Otsu, Japan) to remove any contaminated DNA. The RNA concentration of each sample was measured via spectrophotometry (DeNovix DS-11; Wilmington, DE, USA), equalized, and used for cDNA synthesis with RNA to cDNA EcoDry Premix (Clontech, Mountain View, CA, USA) according to the manufacturer’s protocols. Semi-quantitative reverse transcription polymerase chain reaction (RT-PCR) was performed using gene-specific primers for mouse toll-like receptor-2 (TLR-2), TLR-3, TLR-4, myeloid differentiation primary response 88 (MyD88), tumor necrosis factor-α (TNF-α), interleukin-1α (IL-1α), IL-1β, IL-6, inducible nitric oxide synthase (iNOS), and glyceraldehyde 3-phosphate dehydrogenase (GAPDH) (Table S1). PCR products were run on a 1.5% agarose gel and visualized under ultra-violet light. The expression pattern of each gene was quantified with ImageJ software version 1.52 [16]. Protein levels of released cytokines TNF-α and IL-6 in the supernatants were quantified via enzyme-linked immunosorbent assay (ELISA), using a Mouse Quantikine TNF-α ELISA kit (R&D Systems, Minneapolis, MN, USA) and a Mouse Quantikine IL-6 ELISA kit (R&D Systems, Minneapolis, MN, USA) following the manufacturer’s instructions. BV-2 microglial cells treated with 1 µg/mL of LPS (Sigma, St. Louis, MO, USA) were used as positive controls. BV-2 microglial cells not stimulated with rNfCB or LPS were used as negative controls.

### 2.4. Investigation of MAPK Signaling Pathways in BV-2 Microglial Cells Stimulated by rNfCB

To investigate MAPK signaling pathways involved in the rNfCB-induced immune response of BV-2 microglial cells, the cells were pre-treated with each inhibitor for c-Jun N-terminal kinase (JNK) (SP600125; Calbiochem, San Diego, CA, USA), p38 (SB239063; Calbiochem, San Diego, CA, USA), or extracellular signal-regulated protein kinase (ERK) (U0126; Calbiochem, San Diego, CA, USA) at different concentrations (1 µM or 10 µM) for 3 h. Cells were then stimulated with rNfCB (100 µg/mL) for 6 h and harvested. Total RNA was isolated from the cells, and semi-quantitative RT-PCR was performed for TNF-α, IL-1α, IL-1β, and IL-6, as described above. Quantitative ELISAs for TNF-α and IL-6 were also carried out, as described above. For immunoblot analysis, to analyze the phosphorylation status of the MAPKs, BV-2 microglial cells were cultured with rNfCB (100 µg/mL) for 6 h or 9 h, with or without pre-treatment with each MAPK inhibitor for 3 h. These cells were washed with sterile Tris-buffered saline (TBS, pH 7.4) twice, and lysed with RIPA buffer (Thermo Fisher Scientific, Waltham, MA, USA) containing protease/phosphatase inhibitor cocktail (Thermo Fisher Scientific, Waltham, MA, USA) via repeated freezing–thawing steps. The mixture was centrifuged at 13,000 rpm for 30 min at 4 °C. The supernatant was collected and quantified using a BCA protein assay kit (Thermo Fisher Scientific, Waltham, MA, USA). Protein samples (20 µg each) were analyzed via 12% SDS–PAGE, and then transferred onto nitrocellulose membranes (0.45 µm; GE Healthcare Life Science, Chicago, IL, USA) at 80 V for 70 min on ice. The membranes were blocked with 5% skimmed milk in TBS containing 1% Tween 20 (TBST) at room temperature for 1 h, followed by incubation with anti-β-actin, anti-JNK, anti-p38, anti-ERK, anti-phospho-JNK, anti-phospho-p38, or anti-phospho-ERK monoclonal antibodies (Cell Signaling Technology, Danvers, MA, USA) at 1:1000 dilution in TBST supplemented with 5% bovine serum albumin (BSA) at 4 °C overnight. Membranes were washed with TBST three times (15 min for each wash) and incubated with horseradish peroxidase (HRP)-conjugated anti-rabbit IgG (Sigma, St. Louis, MO, USA) or HRP-conjugated anti-mouse IgG (Sigma, St. Louis, MO, USA) by 1:2000 dilution at room temperature for 2 h. After washing three times with TBST, the membranes were visualized with a SuperSignal Pico PLUS chemiluminescent substrate (Thermo Fisher Scientific, Waltham, MA, USA). β-actin was used as the internal control.

### 2.5. Investigation of NF-κB Involvement in BV-2 Microglial Cells Stimulated by rNfCB

Involvement of NF-κB in the rNfCB-induced immune response of BV-2 microglial cells was analyzed. BV-2 microglial cells were pre-treated with NF-κB inhibitor (MG132; Calbiochem, San Diego, CA, USA) or AP-1 inhibitor (SR11302; Calbiochem, San Diego, CA, USA) at different concentrations (1 µM or 10 µM) for 3 h. The cells were then stimulated with rNfCB (100 µg/mL) for 6 h and harvested. Semi-quantitative RT-PCR for TNF-α, IL-1α, IL-1β, and IL-6 and quantitative ELISAs for TNF-α and IL-6 were performed as described above. To analyze the phosphorylation and nuclear translocation of p65, immunoblotting was conducted. BV-2 microglial cells were cultured with rNfCB (100 µg/mL) in the presence or absence of pre-treatment of MG132 for 6 h and 9 h. The cells were washed with sterile TBS twice. The cytosolic fraction and nuclear fraction were then isolated using an ExKineTM Nuclear Protein Extraction Kit (Abbkine Inc., Wuhan, China), following the manufacturer’s protocols. The protein concentration in each sample was quantified using a BCA protein assay kit (Thermo Fisher Scientific, Waltham, MA, USA). Protein samples (20 µg each) were analyzed via 10% SDS–PAGE and then transferred onto nitrocellulose membranes (0.45 µm; GE Healthcare Life Science, Chicago, IL, USA) at 80 V for 90 min on ice. The membranes were blocked with 5% skimmed milk in TBST for 1 h, followed by incubation with anti-β-actin, anti-Lamin A/C, anti-p65, and anti-phospho-p65 monoclonal antibodies (Cell Signaling Technology, Danvers, MA, USA) at 1:1000 dilution in TBST supplemented with 5% BSA at 4 °C overnight. Membranes were washed with TBST three times (15 min for each wash) and incubated with HRP-conjugated anti-rabbit IgG (Sigma, St. Louis, MO, USA) or HRP-conjugated anti-mouse IgG (Sigma, St. Louis, MO, USA) by 1:2000 dilution at room temperature for 2 h. After washing 3 times with TBST, the membranes were visualized using a SuperSignal Pico PLUS chemiluminescent substrate (Thermo Fisher Scientific, Waltham, MA, USA). β-actin and Lamin A/C were used as internal controls.

### 2.6. Statistical Analysis

All of the above experiments were conducted with three independent replications. Data were presented as the mean ± standard deviation (SD) of three individual assays. Statistical significance was analyzed using a one-way of variance (ANOVA) with Dunnett’s post hoc test. The difference of the mean values was considered as statistically significant at *p* < 0.01.

## 3. Results

### 3.1. rNfCB Does Not Induce Cytotoxicity to BV-2 Microglial Cells

The rNfCB was produced in *E. coli* and purified. SDS–PAGE analysis revealed a high purity of rNfCB (Figure S1a). The purified recombinant protein was refolded and activated to the mature enzyme. Potential contamination by LPS in the protein was depleted. To analyze the potential cytotoxicity of LPS-depleted rNfCB to BV-2 microglial cells, different amounts of rNfCB protein were administered to BV-2 microglial cells, and morphological changes and potential cytotoxicity to cells were analyzed. No significant cytotoxicity was observed when cells were treated with rNfCB at concentrations up to 100 μg/mL (Figure S1b).

### 3.2. rNfCB Induces the Expression of TLR-2, TLR-4, and MyD88 in BV-2 Microglial Cells

**Figure 1:**
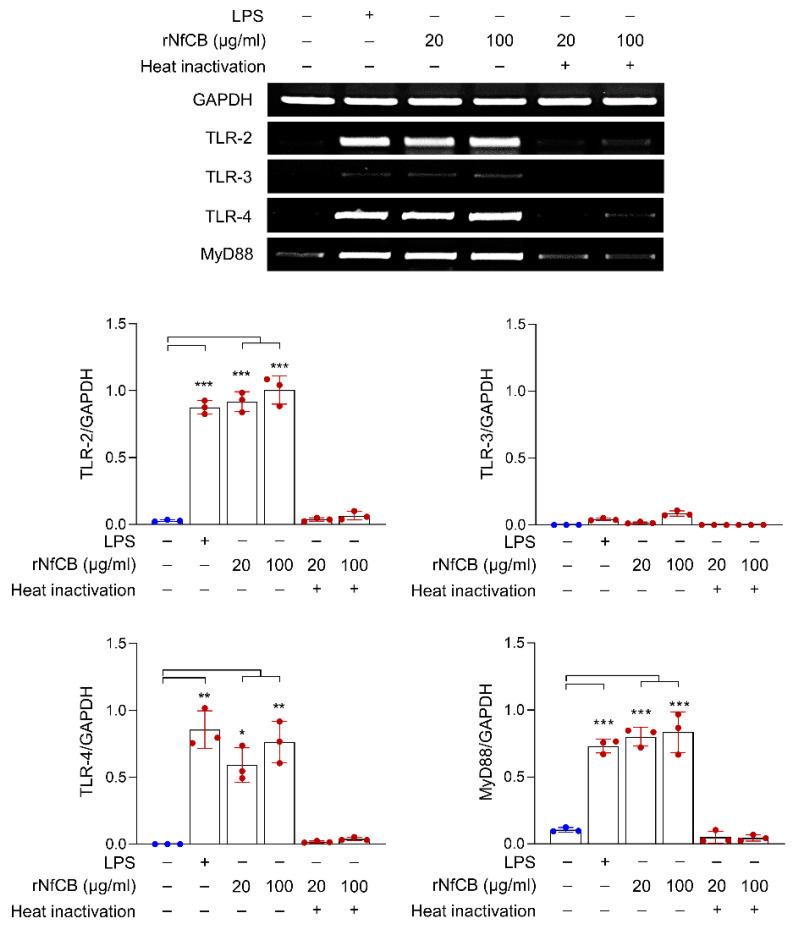
rNfCB activates BV-2 microglial cells via the MyD88-dependent TLR-2/TLR-4 pathway. BV-2 microglial cells were treated with different concentrations (20 µg/mL or 100 µg/mL) of active or heat-inactivated rNfCB. Cells were harvested at 6 h after treatment. Expression levels of TLR-2, TLR-3, and TLR-4, and the downstream adaptor MyD88 were analyzed via semi-quantitative RT-PCR. Bar graphs show the quantitative expression pattern of each gene, analyzed as the fold induction of each gene relative to GAPDH inthree independent experiments. One-way ANOVA with Dunnett’s post hoc test was performed as multiple comparisons with the negative control without treatment with either LPS or rNfCB. *** *p* < 0.0001, ** *p* < 0.001, * *p* < 0.01.

To analyze the initial point of immune response in BV-2 microglial cells induced by rNfCB, the cells were treated with different concentrations of active or heat-inactivated rNfCB. Semi-quantitative RT-PCR analysis revealed that expression levels of TLR-2, TLR-4, and MyD88 were remarkably increased upon treatment with active rNfCB, whereas TLR-3 expression was not greatly affected by the treatment (Figure 1). Meanwhile, heat-inactivated rNfCB did not induce the expression of TLR-2, TLR-4, or MyD88 in BV-2 microglial cells.

### 3.3. rNfCB Induces the Enhanced Production of Pro-Inflammatory Cytokines in BV-2 Microglial Cells

**Figure 2:**
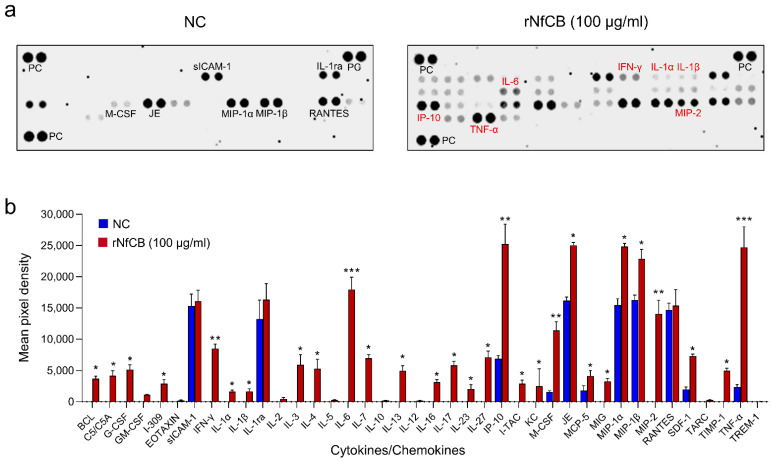
rNfCB induces the production of diverse cytokines/chemokines in BV-2 microglial cells. (**a**) Cytokine array assay. BV-2 microglial cells were stimulated with rNfCB (100 µg/mL). The culture supernatants were harvested and subjected to cytokine array analysis. The culture supernatant from rNfCB-untreated BV-2 microglial cells was used as a negative control (NC). Each cytokine or chemokine is represented as double dots. PC is a positive reference control to verify the reaction. (**b**) Quantitative analysis. The density of dots corresponding to each cytokine or chemokine was analyzed quantitatively, and the value was presented as mean ± SD. One-way ANOVA with Dunnett’s post hoc test was performed as multiple comparisons. *** *p* < 0.0001, ** *p* < 0.001, * *p* < 0.01.

**Figure 3:**
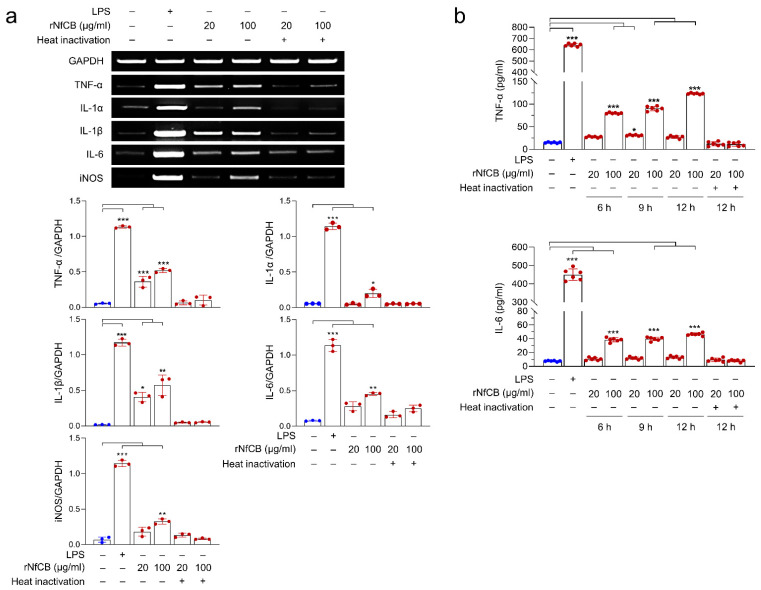
rNfCB induces production of pro-inflammatory cytokines in BV-2 microglial cells. (**a**) mRNA expression. BV-2 microglial cells were treated with different concentrations (20 µg/mL or 100 µg/mL) of active and heat-inactivated rNfCB. Cells were harvested at 6 h after treatment. Semi-quantitative RT-PCR was performed to analyze expression patterns of cytokines (TNF-α, IL1-α, IL-1β, and IL-6) and iNOS. Bar graphs indicate the quantitative expression profile of each gene, represented as -fold induction of each gene relative to GAPDH in three independent experiments. (**b**) Quantitative ELISA. BV-2 microglial cells were treated with different concentrations (20 µg/mL and 100 µg/mL) of rNfCB for different time points (6 h, 9 h, or 12 h). Heat-inactivated rNfCB was administered to the cells for 12 h. At indicated time points, the supernatant was collected and protein levels of TNF-α and IL-6 were analyzed via ELISA. Values were presented as mean ± SD of three independent experiments. One-way ANOVA with Dunnett’s post hoc test was performed as multiple comparisons with the negative control without treatment, with either LPS or rNfCB. *** *p* < 0.0001, ** *p* < 0.001, * *p* < 0.01.

The expression profiles of cytokines and chemokines in BV-2 microglial cells stimulated by rNfCB were analyzed (Figure 2). BV-2 microglial cells stimulated with rNfCB produced diverse pro-inflammatory cytokines and chemokines, particularly TNF-α, IL-6, interferon gamma (IFN-γ), macrophage colony-stimulating factor (M-CSF), macrophage inflammatory protein 2 (MIP-2), and IFN-γ-inducible protein 10 (IP-10). The expressions of IL-1α and IL-1β were also detected. Meanwhile, no or low level of anti-inflammatory cytokines such as IL-4, IL-10, and IL-13 was found. Expression profiles of major pro-inflammatory cytokines were further investigated. Expression levels of pro-inflammatory cytokines, including TNF-α, IL-1α, IL-1β, and IL-6 were increased after treatment with active rNfCB, in a dose-dependent manner (Figure 3a). Expression levels of TNF-α, IL-1β, and IL-6 were increased by both concentrations of rNfCB (20 µg/mL and 100 µg/mL), while IL-1α was only increased by 100 µg/mL of rNfCB. The expression of iNOS was also increased by active rNfCB. Meanwhile, expression levels of these cytokines and iNOS were not greatly affected by treatment with heat-inactivated rNfCB. To further analyze the up-regulated expression of these cytokines at the protein level, ELISAs for TNF-α and IL-6 were performed at different time points (6, 9, and 12 h) after treatment with active rNfCB (Figure 3b). Both cytokines were significantly increased by active rNfCB in dose-dependent and time-dependent manners. Meanwhile, heat-inactivated rNfCB did not induce the production of these cytokines.

### 3.4. Production of Pro-Inflammatory Cytokines Are Mediated by the MAPK Pathway

**Figure 4:**
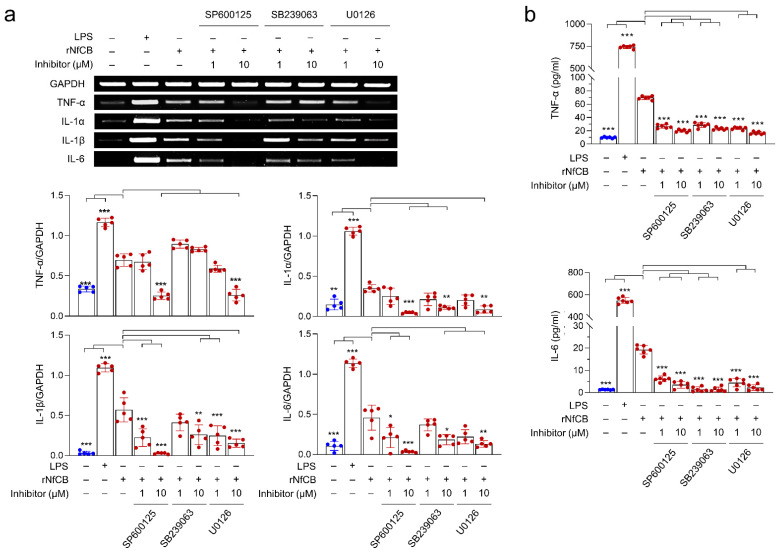
MAPK signaling pathways are involved in pro-inflammatory immune response of BV-2 microglial cells stimulated by rNfCB. (**a**) mRNA expression. BV-2 microglial cells were pre-treated with different concentrations (1 µM or 10 µM) of JNK inhibitor (SP600125), p38 inhibitor (SB239063), or ERK inhibitor (U0126) for 3 h. rNfCB (100 µg/mL) was then administered to the cells. The mRNA expressions of TNF-α, IL-1α, IL-1β, and IL-6 were analyzed via semi-quantitative RT-PCR. Bar graphs indicate the quantitative expression profile of each gene, represented as -fold induction of each gene relative to GAPDH in three independent experiments. (**b**) Quantitative ELISA. Production of TNF-α and IL-6 were measured using ELISA. Values are presented as mean ± SD of three independent experiments. One-way ANOVA with Dunnett’s post hoc test was performed as multiple comparisons with the control, treated with rNfCB. *** *p* < 0.0001, ** *p* < 0.001, * *p* < 0.01.

**Figure 5:**
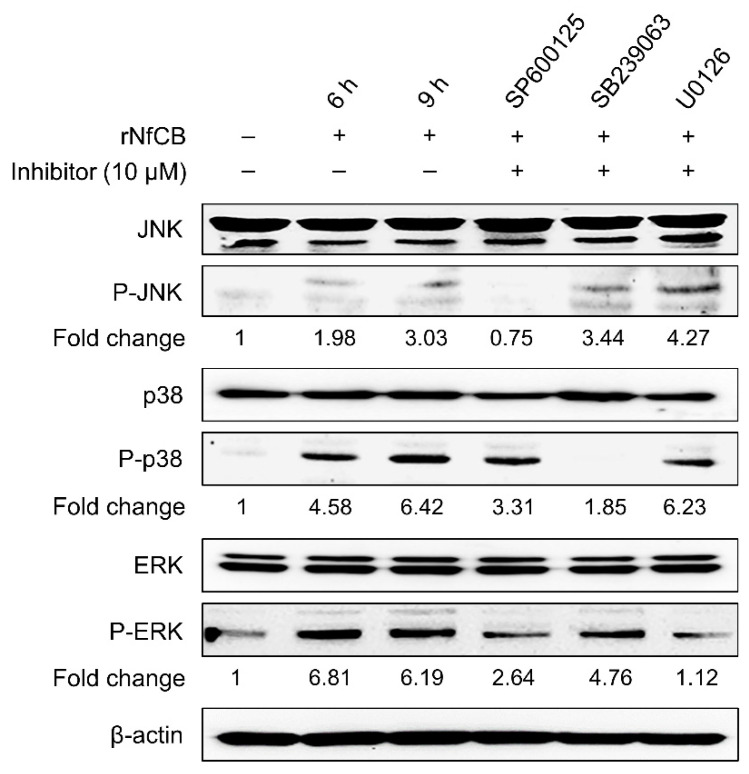
Phosphorylation levels of JNK, p38, and ERK in rNfCB-stimulated BV-2 microglial cells. To analyze phosphorylation levels of MAPKs, BV-2 microglial cells were pre-treated with JNK, p38, or ERK inhibitor, followed by treatment with rNfCB (100 µg/mL). Total proteins were extracted from the cells, and phosphorylation levels of JNK, p38, and ERK were analyzed via immunoblot using a specific antibody for each protein. The total JNK, p38, ERK, and β-actin were used as internal controls. Fold-change means relative density change compared to negative control without treatment with rNfCB and inhibitor.

To examine whether the MAPK signaling pathway was involved in cytokine production in BV-2 microglial cells stimulated by rNfCB, cells were pre-treated with each MAPK inhibitor for JNK (SP600125), p38 (SB239063), or ERK (U0126) at different concentrations (1 µM or 10 µM), and then stimulated with active rNfCB. Overall expression levels of cytokines were decreased when cells were pre-treated with each inhibitor (Figure 4a). Expression levels of IL-1α, IL-1β, and IL-6 were decreased when cells were pre-treated with each inhibitor in a dose-dependent manner. Meanwhile, the expression of TNF-α was not significantly affected by SB239063, a p38 inhibitor, although its expression was significantly decreased after pre-treatment with SP600125 or U0126 (Figure 4a). However, the protein levels of TNF-α and IL-6 were significantly decreased by pre-treatment with JNK, p38, and ERK inhibitors, based on the ELISA (Figure 4b). Phosphorylation levels of JNK, p38, and ERK were also analyzed, to confirm the association of MAPK signaling pathways with the rNfCB-induced pro-inflammation immune response of BV-2 microglial cells. Phosphorylation levels of JNK, p38, and ERK were increased by treatment with active rNfCB in a time-dependent manner (Figure 5). However, the phosphorylation levels of JNK and p38 were significantly abolished after pre-treatment with their corresponding inhibitors, respectively. A minor reduction in ERK phosphorylation was also detected after pre-treatment with an ERK inhibitor.

### 3.5. rNfCB-Induced Pro-Inflammatory Immune Response of BV-2 Microglial Cells Is Regulated via NF-κB and AP-1 Signaling Pathways

**Figure 6:**
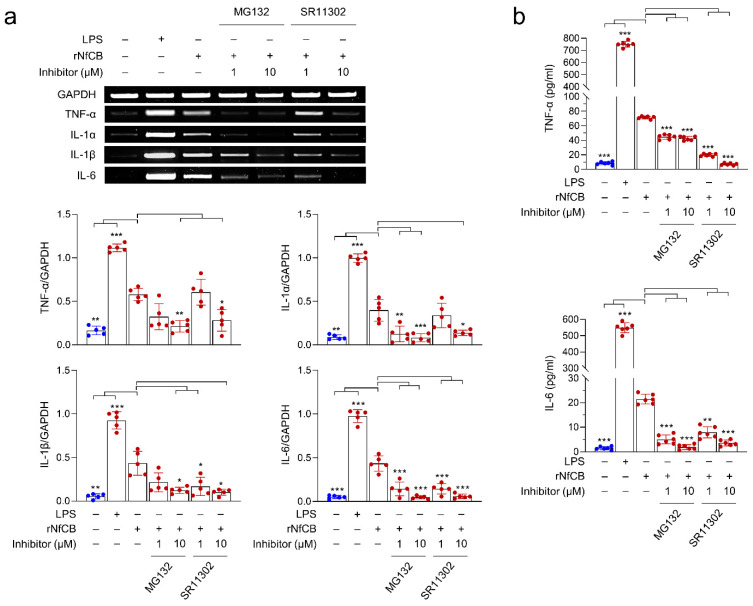
Effects of NF-κB and AP-1 inhibitors on the pro-inflammatory immune response of BV-2 microglial cells stimulated by rNfCB. (**a**) mRNA expression. BV-2 microglial cells were pre-treated with different concentrations (1 µM or 10 µM) of NF-κB inhibitor (MG132) and AP-1 inhibitor (SR11302) for 3 h, followed by treatment with rNfCB (100 µg/mL). mRNA expression levels of TNF-α, IL-1α, IL-1β, and IL-6 were analyzed via semi-quantitative RT-PCR. Bar graphs indicate the quantitative expression profile of each gene, represented as -fold induction of each gene relative to GAPDH in three independent experiments. (**b**) Quantitative ELISA. Production of TNF-α and IL-6 were measured using ELISA. Values are presented as mean ± SD of three independent experiments. One-way ANOVA with Dunnett’s post hoc test was performed as multiple comparisons with the control treated with rNfCB. *** *p* < 0.0001, ** *p* < 0.001, * *p* < 0.01.

**Figure 7:**
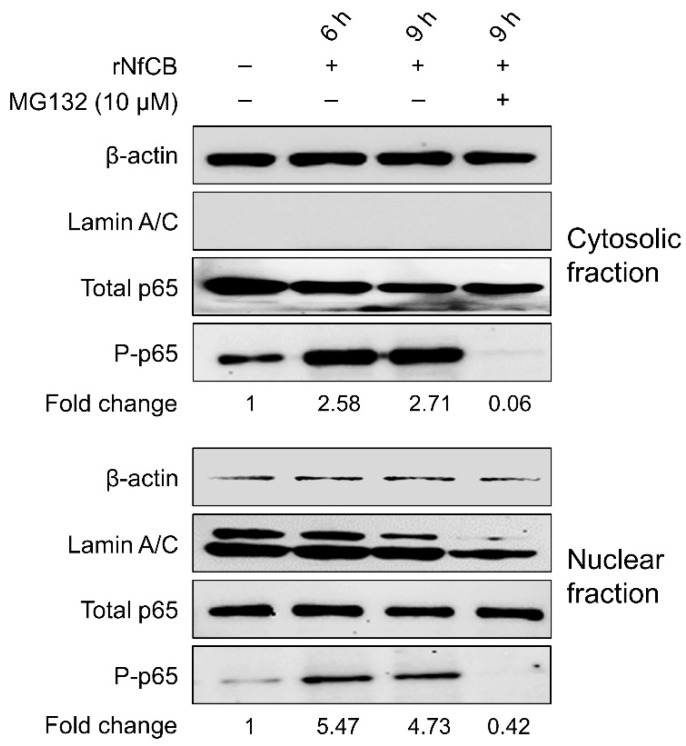
Phosphorylation and nuclear translocation of p65 in rNfCB-stimulated BV-2 microglial cells. To analyze phosphorylation level and translocation of p65, BV-2 microglial cells were treated with rNfCB (100 µg/mL) with or without pre-treatment with MG132. Cytoplasmic proteins and nuclear proteins were extracted from the cells separately. Phosphorylation of p65 was analyzed by immunoblot using a specific antibody for each protein. β-actin and Lamin A/C were used as internal controls. Fold-change means relative density change of P-p65 compared to negative control without treatment of rNfCB and inhibitor.

To investigate whether NF-κB and AP-1 signaling pathways are associated with the rNfCB-induced pro-inflammatory immune response of BV-2 microglial cells, the effects of the NF-κB inhibitor (MG132) and AP-1 inhibitor (SR11302) on cytokine production were analyzed. RT-PCR analysis showed that the expression levels of cytokines were decreased by pre-treatment with MG132 or SR11302 in a dose-dependent manner (Figure 6a). The expression levels of TNF-α, IL-1α, IL-1β, and IL-6 were suppressed by both MG132 and SR11302, although reduced expression levels of these cytokines slightly differed with each inhibitor. Consistent with the patterns of RT-PCR, protein levels of TNF-α and IL-6 were also affected by these inhibitors (Figure 6b). Both TNF-α and IL-6 were significantly decreased by MG132 and SR11302. The phosphorylation level of p65 was increased in both the cytosolic and nuclear fractions of rNfCB-stimulated BV-2 microglial cells. Meanwhile, p65 phosphorylation was significantly inhibited with the pre-treatment of MG132 (Figure 7).

## 4. Discussion

Microglia cells are macrophage-like cells primarily distributed in the brain and CNS. They serve as a front line of immune defense by removing damaged neuronal cells and infections [17]. They are maintained in a resting state under physiological conditions and become activated when they are exposed to pathogens or pathogenic molecules [18,19]. Several studies have been conducted to understand the functional relevance of microglial cells in *N. fowleri* infection [4,7,20,21]. These studies have partially implied the essential immunological functions of microglial cells against *N. fowleri* infection. However, a further understanding of immune responses of microglial cells against *N. fowleri* infection is necessary to gain insights into the pathogenic mechanisms of this parasite and PAM.

*N. fowleri* expresses several cathepsin B family cysteine proteases [15]. Their expression levels are high at the actively proliferating trophozoite stage, and they are secreted or released from the amoeba. The primary biological functions of these enzymes are likely to be associated with host tissue invasion and host cell destruction, as these enzymes can hydrolyze cellular structural proteins such as collagen and fibronectin [15]. The partial hydrolysis of the immunoglobulins A, G, and M also suggests their potential roles in host immune evasion [15]. Host immune modulation by cysteine proteases from protozoan parasites has been studied. Secreted cysteine proteases of *Entamoeba histolytica* can stimulate human mast cells, resulting in the production of IL-8 via a protease activated receptor 2 (PAR2)-independent mechanism [22]. *Giardia lamblia* can impair the LPS-evoked pro-inflammatory response in macrophages via the inhibition of cyclooxygenase-2 and iNOS expression. Proteases of the parasite, including cysteine proteases, might participate in this process [23].

In this study, the immune response of BV-2 microglial cells evoked by rNfCB was analyzed to understand the underlying molecular and pathological roles of the enzyme associated with PAM. It was found that rNfCB enhanced the expression levels of TLR-2, TLR-4, and MyD88 in BV-2 microglial cells. TLRs are pattern-recognition receptors that play central roles in the initiation of the immune response [24]. The expression of TLRs is a critical determinant of inflammatory responses, as well as specific downstream intracellular signaling cascades. The function of the canonical TLR-4 pathway has been demonstrated in a previous study, showing that mucoepithelial cells can produce pro-inflammatory cytokines and chemokines such as TNF-α, IL-1β, and IL-8 when they are exposed to *N. fowleri* trophozoites [25]. The up-regulation of expression levels of TLR-2, TLR-4, and MyD88 in BV-2 microglial cells stimulated by rNfCB suggests that NfCB can evoke initial inflammation responses in BV-2 microglial cells via the MyD88-dependent TLR-2/TLR-4 pathway. BV-2 microglial cells stimulated by rNfCB showed increased mRNA and protein levels of pro-inflammatory cytokines, including TNF-α, IL-1α, IL-1β, and IL-6, suggesting that rNfCB could induce a pro-inflammatory immune response in cells. The increased phosphorylation of MAPK proteins, including JNK, p38, and ERK in BV-2 microglial cells, and the enhanced expression levels of TNF-α, IL-1α, IL-1β, and IL-6 by rNfCB were effectively suppressed by inhibitors for JNK, p38, and ERK, suggesting the association of MAPK with rNfCB induced a pro-inflammatory immune response in cells. Expression levels of these cytokines were also down-regulated by NF-κB and AP-1 inhibitors. The rNfCB also enhanced the phosphorylation and nuclear translocation of p65, a key element of NF-κB, in BV-2 microglial cells. Such enhancements were effectively inhibited by MG132. These results collectively suggest that NfCB can evoke pro-inflammatory immune responses in BV-2 microglial cells, characterized by enhanced expression levels of TNF-α, IL-1α, IL-1β, and IL-6 via the NF-κB and AP-1-dependent MAPK pathways.

Interestingly, heat-inactivated rNfCB did not induce inflammatory responses in BV-2 microglial cells. This suggests that proteolytic activity or the intact structural conformation of NfCB is critical to inducing inflammatory responses in cells. Several previous studies have indicated the importance of enzyme activity of parasite cysteine proteases in host immune modulation. A cysteine protease of *Leishmania mexicana* can induce Th2 immune responses in the host, whereas the inactivated enzyme fails to induce such responses [26]. The inactivation of the cysteine protease of *E. histolytica* causes a significant reduction in IL-8 production in human mast cells [22]. The functional significance of the structural intactness and enzymatic activity of parasite cysteine proteases relevant to the host immune response remains unclear. The underlying mechanism should be elucidated further.

## 5. Conclusions

**Figure 8:**
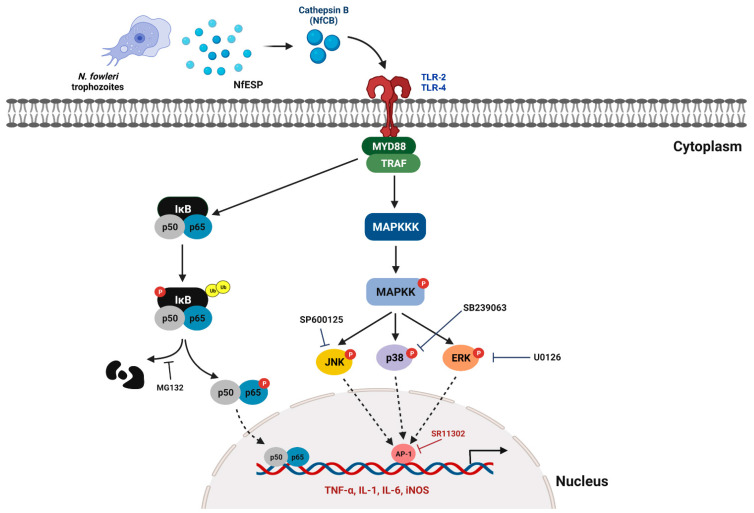
Summarized scheme of signaling pathways involved in rNfCB-induced pro-inflammatory immune responses of BV-2 microglial cells. NfCB induces a pro-inflammatory immune response of BV-2 microglial cells, which is initiated via MyD88-dependent TLR-2/TLR-4 pathway and mediated by NF-κB- and AP-1-dependent MAPK signaling pathways. The image was created with BioRender (https://biorender.com, accessed on 25 May 2022). NfESP, excretory and secretory products of *N. fowleri*.

NfCB can initiate pro-inflammatory responses in BV-2 microglial cells via the MyD88-dependent TLR-2/TLR-4 pathway. The enhanced expression of pro-inflammatory cytokines such as TNF-α, IL-α, IL-1β, and IL-6 is mediated by NF-κB and the AP-1-dependent MAPK signaling pathway (Figure 8). NfCB may contribute to the pathogenesis of PAM by inducing a pro-inflammatory immune response in microglial cells via a contact-independent pathway, which may promote subsequent inflammatory cascades in the brain. The enhanced expression of pro-inflammatory cytokines in microglial cells may exacerbate deleterious inflammations in *N. fowleri*-infected foci, and accelerate inflammatory cascades by activating other types of cells such as glial cells and astrocytes in the brain. Considering that NfCB hydrolyzes cellular structural proteins with potent cytotoxicity to BV-2 microglial cells at high concentrations, this protein may also be involved in the direct cellular damage of brain cells. This study expands our knowledge of the immune response of microglial cells against *N. fowleri* infection, as well as the pathogenic mechanisms of NfCB.

## Data Availability

The data supporting the conclusions of this article are provided within the article and its additional files. The original datasets analyzed in the current study are available from the corresponding author upon request.

## Supplementary Materials

The following supporting information can be downloaded at: https://www.mdpi.com/article/10.3390/ijms23158388/s1.
