# Supplementary material for: Naegleria fowleri Cathepsin B Induces a Pro-Inflammatory Immune Response in BV-2 Microglial Cells via NF-κB and AP-1 Dependent-MAPK Signaling Pathway
Source: Int J Mol Sci. 2022 Jul 29;23(15):8388. doi: 10.3390/ijms23158388 (PMC9369353; doi:10.3390/ijms23158388)
Supplement: Supplementary file 1 [file ijms-23-08388-s001.zip › Supplementary Figure S1.pdf]

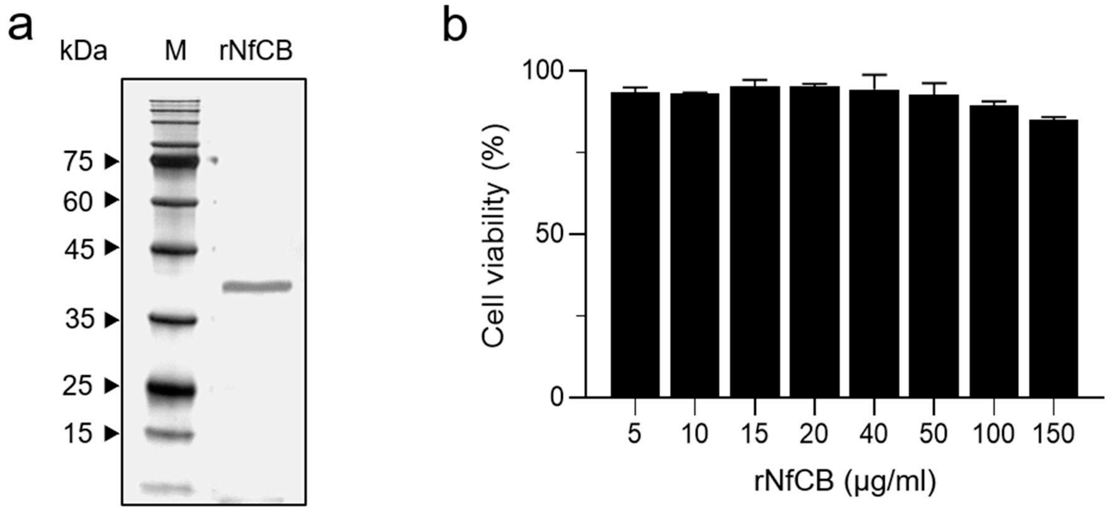

Supplement Figure S1. Production of rNfCB and its cytotoxicity to BV-2 microglial cells. (**a**) Purification of rNfCB. The recombinant protein was produced in *E. coli*, purified, and analyzed by 12% SDS–PAGE. Lane M, Size marker proteins, lane rNfCB, purified rNfCB. (**b**) Cytotoxicity assay. Different concentration of LPS-depleted rNfCB (0, 5, 10, 15, 20, 40, 50, 100, or 150 μg/ml) was treated to BV-2 microglial cells and incubated for 24 h. Cell viability was measured using CellTiter-Blue® Cell Viability Assay (Promega). Percentage of cell viability was calculated by comparing each value to negative control without treatment of rNfCB (100%). Values were presented as mean ± standard deviation of three independent assays.
