# Supplementary material for: Naegleria fowleri Cathepsin B Induces a Pro-Inflammatory Immune Response in BV-2 Microglial Cells via NF-κB and AP-1 Dependent-MAPK Signaling Pathway
Source: Int J Mol Sci. 2022 Jul 29;23(15):8388. doi: 10.3390/ijms23158388 (PMC9369353; doi:10.3390/ijms23158388)
Supplement: Supplementary file 1 [file ijms-23-08388-s001.zip › Supplementary Table S1.pdf]

Table S1. **Primer sequences used in the study**

Gene	Primer sequences
TLR-2	Forward: 5′-TGCTTTCCTGCTGGAGATTT -3′<br>Reverse: 5′-TGTAACGCAACAGCTTCAGG-3′
TLR-3	Forward: 5′-CCCCCTTTGAACTCCTCTTC-3′<br>Reverse: 5′-TTTCGGCTTCTTTTGATGCT-3′
TLR-4	Forward: 5′-GCATGGCTTACACCACCTCT-3′<br>Reverse: 5′-GTGCTGAAAATCCAGGTGCT-3′
MyD88	Forward: 5′-GCTGCTGGCCTTGTTAGACCGTGA-3′<br>Reverse: 5′-GACGTCACGGTCGGACACACACAAC-3′
TNF-α	Forward: 5′-CATCCTCTCAAAATTCGAGTGACA-3′<br>Reverse: 5′-TGGGAGTAGACAAGGTACAACCC-3′
IL-1α	Forward: 5'-ATGGCCAAAGTTCCTGACTT-3'r<br>Reverse: 5'-TGGTCTTCTCCTTGAGCGCT-3'
IL-1β	Forward: 5′-TGCAGAGTTCCCCAACTGGTACAT-3′<br>Reverse: 5′-GTGCTGCCTAATGTCCCCTTGAAT-3′
IL-6	Forward: 5′-CCGGAGAGGAGACTTCACAG-3′<br>Reverse: 5′-GGAAATTGGGGTAGGAAGGA-3′
iNOS	Forward: 5′-AATGGCAACATCAGGTCGGCCATCACT-3′<br>Reverse: 5′-GCTGTGTGTCACAGAAGTCTCGAACTC-3′
GAPDH	Forward: 5′-ACCAGAGTCCATGCCATCAC-3′<br>Reverse: 5′-CACCACCCTGTTGCTGTAGCC-3′
